# Editorial: The pharmacological effects and mechanisms of drugs against human diseases by modulating redox homeostasis - volume II

**DOI:** 10.3389/fphar.2025.1665486

**Published:** 2025-08-08

**Authors:** Xiaofang Zhang, Ming Xu, Zhihao Liu, Luchen Shan

**Affiliations:** ^1^ Academician Collaborative Laboratory for Basic Research and Translation of Chronic Diseases, Central Laboratories, The First Affiliated Hospital of Jinan University, Jinan University, Guangzhou, China; ^2^ Guangdong Provincial Key Laboratory for Chronic Disease Interactive Network Research, Jinan University, Guangzhou, China; ^3^ School of Data Science, Shimonoseki City University, Shimonoseki, Japan; ^4^ Resolian, Malvern, PA, United States; ^5^ State Key Laboratory of Bioactive Molecules and Druggability Assessment, International Cooperative Laboratory of Traditional Chinese Medicine Modernization and Innovative Drug Development of Ministry of Education (MOE), College of Pharmacy, Jinan University, Guangzhou, China

**Keywords:** reactive oxygen species (ROS), oxidatie stress, disease, homeostasis, drugs

Once widely promoted by journal articles, textbooks, and the media for their presumed health benefits, antioxidants are now understood within a more nuanced framework. It is increasingly recognized that oxidants—particularly reactive oxygen species (ROS)—play dual roles as both harmful agents and essential signaling molecules (Sies et al., 2022). While excessive ROS can cause oxidative stress and damage to DNA, proteins, and lipids, contributing to various diseases, low to moderate levels are crucial for maintaining normal cellular functions (Sies, 2021). Reflecting this conceptual shift, the present Research Topic focuses on redox homeostasis modulators rather than narrowly defined antioxidants

The seven original research articles and two literature reviews published in this Research Topic illustrate the evolving conceptual framework from three distinct perspectives:

Inhibiting ROS overproduction and accumulation confers tissue protection. Mao et al. demonstrated that acrolein—an environmental pollutant and a byproduct of lipid peroxidation (a reactive molecule)—induces glutathione (GSH) depletion, triggering Sertoli cell ferroptosis and lipid peroxidation, ultimately leading to male reproductive toxicity and cyclophosphamide (a precursor of acrolein)-induced cystitis (Mao et al.; Mao et al.). Enhancing endogenous hydrogen sulfide (H_2_S) production or administering exogenous H_2_S counteracts this process by directly neutralizing acrolein to restore glutathione levels and suppress oxidative signaling. Another study by Zhu et al. demonstrated that gallic acid mitigates intervertebral disc degeneration by upregulating the antioxidant enzyme GPX2 and the transcription factor NRF2 (coordinating the cellular antioxidant response) while reducing levels of 4-hydroxynonenal—a byproduct of lipid peroxidation—and mitochondrial iron deposition (Zhu et al.). Lu et al. revealed that D-mannose enhances antioxidant defenses in ulcerative colitis models by increasing the levels of superoxide dismutase and glutathione while reducing malondialdehyde, a marker of lipid peroxidation (Lu et al.). In a related study, Yan et al. reported that dl-3-n-butylphthalide disrupts Keap1-Nrf2 complex formation, thereby preventing Nrf2 ubiquitination, promoting the expression of antioxidant genes, reducing mitochondrial ROS, and ultimately improving cardiac function in a mouse model of doxorubicin-induced cardiotoxicity (Yan et al.). Hao et al. documented a notable finding: quercetin-encapsulated PEG-PCL micelles (Que-PEG-PCL) significantly attenuate cisplatin-induced nephrotoxicity, reducing serum creatinine and blood urea nitrogen (BUN) levels to 42% and 38%, respectively (Hao et al.). This protective effect is attributed to a reduction in oxidative stress and an enhancement of antioxidant activity. Furthermore, co-administration with Que-PEG-PCL increased cisplatin plasma exposure to 323% while decreasing renal clearance to 14%, likely through inhibition of the organic cation transporter 2 (OCT2). In a CT26 syngeneic tumor model, this combination therapy suppressed tumor volume by 84% compared to the control group.

Leveraging ROS generation enables precision therapies. Pang et al. showed that the iridium(III) complex Ir-1 enhances lung cancer radiosensitivity by increasing intracellular ROS, disrupting mitochondrial membrane potential, and activating the Bax/Bak–cytochrome c–caspase-9 apoptotic pathway, leading to G2/M arrest and apoptosis with minimal toxicity to normal fibroblasts (Pang et al.). Similarly, the nanocarrier Que-PEG-PCL boosts cisplatin’s pro-oxidant effects in CT26 colon cancer, synergistically increasing ROS, triggering mitochondrial depolarization, and inducing apoptosis—achieving 84% tumor suppression, far surpassing free quercetin or cisplatin alone. Together, these strategies illustrate the “double-edged sword” of redox modulation: using ROS to selectively eliminate tumors while sparing healthy tissue.

Natural compounds as pleiotropic regulators of redox homeostasis. While the research articles in this Research Topic present new findings on the context-dependent benefits of both antioxidants and pro-oxidants, two accompanying review articles offer a broader and more comprehensive perspective on antioxidants derived from natural sources. Zhang et al. synthesized current knowledge on the antioxidative properties and therapeutic effects of flavonoids in mitigating vasospasm associated with myocardial and cerebral infarction (Zhang et al.). Meanwhile, Wei et al. focused on curcumin, highlighting its dual role in scavenging free radicals and suppressing NF-κB signaling in models of Alzheimer’s disease and dopaminergic neuron loss in Parkinson’s disease (Wei et al.).

This Research Topic of articles in the present Research Topic offers multiple lines of evidence supporting the rationale and therapeutic potential of both antioxidants and pro-oxidants as modulators of redox homeostasis. However, the current studies primarily focus on the isolated effects of these agents, rather than addressing the broader and more critical challenge: the establishment, restoration, and long-term maintenance of redox balance.

The clinical efficacy of antioxidant**s** continues to be a subject of intense debate, with studies yielding conflicting results across both experimental and real-world settings. This ongoing controversy arises from a complex web of biological variability, methodological limitations, and the dual—sometimes paradoxical—roles of reactive oxygen species (ROS) in both physiology and pathology.

Several fundamental questions remain unresolved and point toward promising avenues for future research:

What is redox homeostasis in quantitative terms? To what extent should ROS be reduced or enhanced to confer therapeutic benefit? While, in theory, redox homeostasis can be quantified using molecular markers and redox couples that reflect the balance between oxidizing and reducing species in cells, tissues, or fluids, the practical application of this concept is fraught with challenges. The inherent complexity of biological systems, the transient nature of redox reactions, and the highly context-dependent behavior of both oxidants and antioxidants complicate efforts to measure and modulate redox states precisely.

How can we control the context-dependent effects of antioxidants? The efficacy of antioxidants is not universal. It depends on the timing of administration, the disease context, and individual biological variability. While they may offer benefits in conditions marked by pathological oxidative stress, they could be ineffective—or even harmful—when administered in settings of physiological ROS signaling.

How can we address the complex interplay between ROS and other biological pathways? Oxidative stress rarely acts in isolation. It intersects with numerous other disease mechanisms, such as inflammation, mitochondrial dysfunction, and immune dysregulation. Antioxidants alone may not be sufficient to address these intricate, multifactorial networks.

How should we interpret and manage the antioxidant paradox? Some compounds traditionally regarded as antioxidants can, under certain conditions (e.g., high concentrations or presence of transition metals), exert pro-oxidant effects. This dual nature complicates their use and calls for a deeper understanding of dose-dependent behavior and cellular context.

How can we rationally apply antioxidants and pro-oxidants across disease types? The role of ROS varies widely between diseases. For example, in oncology, ROS-inducing therapies are often necessary to eliminate cancer cells, whereas in cardiovascular or neurodegenerative diseases, reducing oxidative stress might be more beneficial. Thus, redox-based therapies must be tailored to the specific pathophysiological landscape of each condition.
